# Decadal Trend in Agricultural Abandonment and Woodland Expansion in an Agro-Pastoral Transition Band in Northern China

**DOI:** 10.1371/journal.pone.0142113

**Published:** 2015-11-12

**Authors:** Chao Wang, Qiong Gao, Xian Wang, Mei Yu

**Affiliations:** Department of Environmental Sciences, University of Puerto Rico, Rio Piedras, San Juan, Puerto Rico, United States of America; Chinese Academy of Forestry, CHINA

## Abstract

Land use land cover (LULC) changes frequently in ecotones due to the large climate and soil gradients, and complex landscape composition and configuration. Accurate mapping of LULC changes in ecotones is of great importance for assessment of ecosystem functions/services and policy-decision support. Decadal or sub-decadal mapping of LULC provides scenarios for modeling biogeochemical processes and their feedbacks to climate, and evaluating effectiveness of land-use policies, e.g. forest conversion. However, it remains a great challenge to produce reliable LULC maps in moderate resolution and to evaluate their uncertainties over large areas with complex landscapes. In this study we developed a robust LULC classification system using multiple classifiers based on MODIS (Moderate Resolution Imaging Spectroradiometer) data and posterior data fusion. Not only does the system create LULC maps with high statistical accuracy, but also it provides pixel-level uncertainties that are essential for subsequent analyses and applications. We applied the classification system to the Agro-pasture transition band in northern China (APTBNC) to detect the decadal changes in LULC during 2003–2013 and evaluated the effectiveness of the implementation of major Key Forestry Programs (KFPs). In our study, the random forest (RF), support vector machine (SVM), and weighted k-nearest neighbors (WKNN) classifiers outperformed the artificial neural networks (ANN) and naive Bayes (NB) in terms of high classification accuracy and low sensitivity to training sample size. The Bayesian-average data fusion based on the results of RF, SVM, and WKNN achieved the 87.5% Kappa statistics, higher than any individual classifiers and the majority-vote integration. The pixel-level uncertainty map agreed with the traditional accuracy assessment. However, it conveys spatial variation of uncertainty. Specifically, it pinpoints the southwestern area of APTBNC has higher uncertainty than other part of the region, and the open shrubland is likely to be misclassified to the bare ground in some locations. Forests, closed shrublands, and grasslands in APTBNC expanded by 23%, 50%, and 9%, respectively, during 2003–2013. The expansion of these land cover types is compensated with the shrinkages in croplands (20%), bare ground (15%), and open shrublands (30%). The significant decline in agricultural lands is primarily attributed to the KFPs implemented in the end of last century and the nationwide urbanization in recent decade. The increased coverage of grass and woody plants would largely reduce soil erosion, improve mitigation of climate change, and enhance carbon sequestration in this region.

## Introduction

Complex human-nature interactions altered global environments [1,2]. Among these interactions, land use and land cover (LULC) changes significantly [3,4,5], which has important implications on global hydrological and biogeochemical cycles, biodiversity, ecosystem services, and disturbance regimes [6,7,8,9,10].

Prompt and reliable mapping of LULC [11,12,13,14] is essential in the continuous monitoring of land use and land cover change (LULCC) through time, such as forest transition and urban sprawl [4,15,16]. It also provides time series of LULC scenarios for subsequent modeling of biogeochemical and hydrological processes [17,18,19], assessment of ecosystem services [7,9,20,21], and simulation of LULCC feedbacks to regional climate [22].

Remote sensing images with various spectral and spatiotemporal resolutions have become easy to access, especially in the past 10 years, and have been the most important data sources for mapping LULCC due to their spatially continuous information of land surface and high consistence across a range of spatiotemporal scales [12,15]. On the other hand, various classifiers, such as random forest (RF), support vector machine (SVM), and weighted k-nearest neighbors (WKNN), have been applied to detect LULCC across scales from local to globe for various themes, such us urbanization, agricultural abandonment, and forest conversion [15,23,24,25,26].

The random forest classifier uses training data set with multiple feature variables to ‘grow’ designated number of trees, each of which defines the classes from a randomly selected subset of feature variables. Application of the classifier is determined by the proportion of votes by these trees so that the class with greatest voting proportion is determined [27]. Unlike RF, supported vector machine classifier seeks to construct hyperplanes in the feature space so that the distance between classes can be maximized [28]. Compared to RF and SVM, weighed k-nearest neighbors determines the class by the relative frequency of the training data set within a designated number of nearest neighbors k. These classifiers have their own strengths in dealing with multidimensional remote sensing data, nonlinearity between spectral bands, and specific LULC types, therefore, may provide complementary information in mapping LULCC.

There are always error and uncertainty involved in mapping LULC based on remote sensing images. The source of error and uncertainty might include inadequate representation of LULC classes in the training data, insufficient predictor variables, overlapping of spectral characteristics among LULC classes, and weakness of classifiers [24,29]. Although high accuracy of classification has been achieved in many LULC mapping studies, few of them provides in-depth uncertainty information, for example, spatial variation in uncertainty or pixel-level uncertainty. The detailed uncertainty information is essential for application and analysis of the mapped LULC, and is especially important for large area with great spatial heterogeneity of land use, such as ecotones.

As an ecotone supporting tens of million population, the Agro-pasture transition band in northern China (APTBNC) has experienced significant historical and recent environmental changes, such as severe soil erosion, grassland degradation, desertification, and biodiversity loss [30,31,32,33]. Irrational land use, e.g. intensive farming in areas with inappropriate geomorphological, climatic, and soil conditions and livestock overgrazing, is believed to be the primary causes for the ecosystem deterioration [34,35]. The great environmental gradient in ecotones implies the ecosystems and land use are sensitive to shift in climate, and frequent change in land use has been documented in history [36]. The frequent changes in LULC, and the great spatial gradients created the complex landscapes and the challenge in mapping LULCC in APTBNC, hence necessitate robust strategy to reliable LULC maps.

Since 1998, the Chinese government has implemented a series of policies and programs to restore ecosystems and to improve environmental protection. One of them is the “Grain for Green” program [35,37] that calls for converting previously irrationally allocated croplands to forest, shrublands, and grasslands. The effectiveness of these policies and programs deserve objective and quantitative evaluation, so that the concurrent policies and regulations can be correctly adjusted and new ones can be precisely directed.

The objectives of this study are: a) to develop a robust strategy to produce reliable LULC maps for ecotones such as APTBNC based on multiple classifiers and posterior data fusion; b) to provide pixel-level uncertainty to assess spatial variations in classification accuracy; and c) to detect the LULCC in 2003–2013 and evaluate the effectiveness of the land use policies implemented in APTBNC.

## Methodology

### Study area

**Fig 1:**
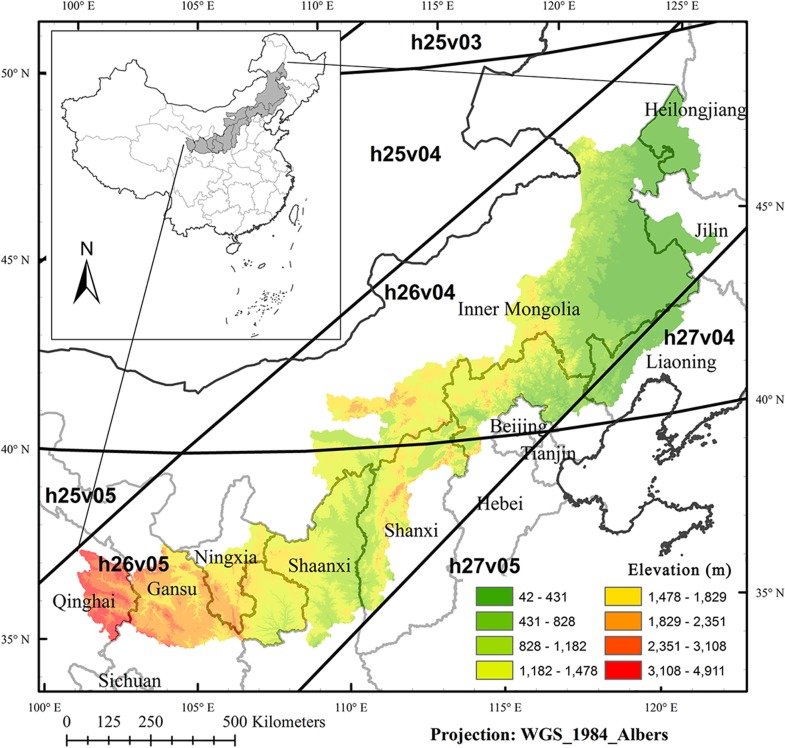
Location of the Agro-pasture transition band in northern China (colored according to the elevation legend). The grids overlaid are the corresponding MODIS tiles (e.g. h26v05).

The Agro-pasture transition band in northern China (APTBNC, ranging over 34.7°-48.6°N and 100.8°-124.8°E with elevation varying from 42 to 4911 m, Fig 1) is an interlaced zone changing from agricultural cultivation in the middle and the southeast to livestock grazing in the northwest [13,32]. With the total area of 724,766 km^2^, APTBNC supports 67 million people in 205 counties across 10 provinces [38]. The climate varies from monsoon climate in the southeast to continental climate in the northwest, resulting a sharp moisture gradient from semi-humid climate with mean annual precipitation (MAP) of 580 mm to semiarid climate with MAP less than 200 mm. Annual mean temperature decreases from 14°C in the south to less than -1°C in the north (1959–2001) [38]. Mostly driven by the patterns of moisture and temperature, large proportions of shrubs and grasses are distributed in the northwest and deciduous broadleaved forests are in the southeast and the north. Agricultural lands lie in the middle and the southeast.

### Datasets for classification

The Moderate Resolution Imaging Spectroradiometer (MODIS) sensors on board the Terra and the Aqua platforms provide reflectance information across wavelengths of 0.4–14.4 μm for monitoring LULCC at the spatial resolution of 250 m– 1 km (http://modis-land.gsfc.nasa.gov/). In this study, the MODIS reflectances and Vegetation Indices (VIs), as well as the phenological parameters derived from the MODIS data, were used as the main data source to map the LULC. We also included topographic information, such as elevation, slope, and aspect, as auxiliary data due to their important roles in vegetation distribution.

Specifically we used the MODIS VI product (MOD13Q1, Collection 5) which includes Normalized Difference Vegetation Index (NDVI), Enhanced Vegetation Index (EVI), blue, red, near infrared (NIR), mid-infrared (MIR) reflectances, and pixel quality assurance (QA) [39,40]. NDVI is derived from the reflectance at red and NIR bands, while EVI further incorporates the reflectance at blue band to correct for residual aerosol effects and also aims to improve its sensitivity to high biomass [39]. The gridded level-3 product of the datasets has the time interval of 16 days and the spatial resolution of about 250 m. Our study area of APTBNC is covered by the MODIS tiles of H26V04, H26V05, H27V04, and H27V05 (Fig 1). We calculated the growing-season statistics of mean, standard deviation, minimum, maximum, and range of the reflectances and VIs and used them as predicates for further classification.

We then extracted phenological metrics from the time series of MODIS EVI with the aid of the free software TIMESAT (version 3.1) [41,42]. TIMESAT applies various smoothing functions, e.g. polynomial, harmonic, or asymmetric Gaussian functions, to approximate upper-envelop seasonal changes imbedded in the satellite VI series, and therefore to reduce the influence of signal noise in the raw data on phenology pattern. By incorporating VI and QA series of a target year and adjacent years, the program will apply QA-adjusted weights and derive phenological parameters in the growing season of the target year. Among the TIMESAT derived parameters, we used the starting date, mid-season date, growing season length, base, peak, and amplitude of VI, rates of increase at the beginning and decrease at the end of a season, and seasonal integrals of VI for further classification analyses [42]. The QA-adjusted weights are full weight for QA = 0 (good), half weight for QA = 1–2 (marginal), and minimal weight of 0.1 for QA = 3 (with clouds).

We used the Shuttle Radar Topography Mission (SRTM) Digital Elevation Model (DEM) at the spatial resolution of about 90 m, with missing data filled by the Consultative Group for International Agriculture Research (CGIAR, SRTM 90m DEM Version 4, http://www.srtm.csi.cgiar.org) [43]. The datasets of DEM and derived slope and aspect were projected and resampled to match the MODIS-derived datasets using a bilinear interpolation method.

### Classification scheme and ground reference data collection

Based on our previous studies in this region we chose: urban area (URBN), agricultural land (AGRI), deciduous broad-leaved (DBLF) and evergreen needle-leaved forests (ENLF), open (OPSH) and closed shrublands (CLSH), grassland (GRAS), bare ground (BARE), and water (WATR), as the LULC classes to be recognized by our classification system [12,38,44].

We applied a holistic strategy to collect ground reference data, which includes the historical vegetation maps, the Google Earth high-resolution imageries (http://www.earth.google.com), Flora of China (http://www.eflora.cn/), and the field observations. First we used stratified random sampling to create random points in each vegetation zone based on the vegetation maps at 1:1,000,000 scales [45]. We then discarded those points that do not have high-resolution images available at Google Earth. By overlaying the MODIS grids, we further removed those points that fall into spatial heterogeneous areas in the target and immediate neighborhood MODIS grids. Based on the high-resolution Google Earth images and the distribution information of dominant species available at Flora of China, we identified ground LULC types for the remaining points according to the visual interpretation schema in literature [25,46,47,48,49]. Ground observations include those available at the field stations of Chinese Ecological Research Network (www.cern.ac.cn). This type of ground reference data collection is suitable for MODIS datasets at the regional scale [25].

### Data mining approach and framework

**Fig 2:**
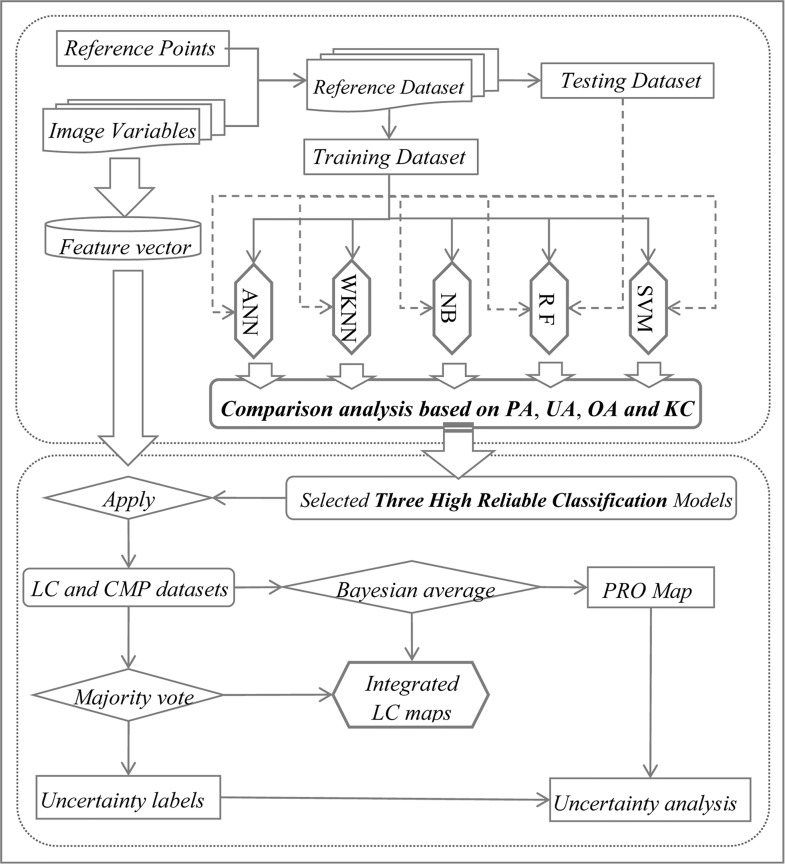
The scheme of data mining procedures for LULC classification. (PA, producer’s accuracy; UA, user’s accuracy; OA, overall accuracy; KC, Kappa coefficient; LC, land cover; CMP, class membership probability; and PRO, probability).

The framework includes three parts (Fig 2): 1) Classification using five commonly-used classifiers, which are artificial neural network (ANN) [50,51], weighted k-nearest neighbors (WKNN) [52,53], naive Bayes (NB) [54,55], Random Forest (RF) [11,25,27,56,57,58,59], and support vector machine (SVM) [28,54,60,61]; 2) Integrated classification based on the results and corresponding uncertainty analysis; 3) LULCC analysis. The classification was carried out by using the packages of nnet, e1071, randomForest, and kknn of the open source statistical software R v. 3.1.1 [62]. We set up the parameters of each classifier by using tuning functions to achieve good performance [63,64]. Details of the parameters of five classifiers used were listed in S1 Table.

#### Classification and comparison of five classifiers

We first extracted the abovementioned statistics of reflectances and VIs, phenological parameters, and topographical information for the sampled points, which consist of 1,947 points of AGRI, 652 of GRAS, 997 of CLSH, 267 of OPSH, 653 of DBLF, 186 of ENLF, 548 of URBN, 248 of BARE, and 148 of WATR. Then we randomly split the sampled data into two datasets, training (70% of points per LULC class) and testing (30% per class). For each of the five classifiers, we used three different combinations of variables for the training data as inputs: C1, growing-season statistics of reflectances (blue, red, NIR, and MIR) and VIs (NDVI and EVI), and topographical information (elevation, slope, and aspect); C2, phenological parameters and topographical information; and C3, growing-season statistics of reflectances and VIs, phenological parameters, and topographical information. Therefore, we have 15 resultant models (5 classifiers X 3 input combinations). We then quantified classification accuracy on the test data using overall classification accuracy, producer’s accuracy, user’s accuracy, and Kappa statistics.

In addition to accuracy, robustness is another key point in comparing classifiers. We analyzed the sensitivity of each classifier to the sample size of training dataset via the following strategy: 1) we created ten training subsets with 10% to 100% of the samples per class randomly selected from the training data, in 10% increments; 2) for each of the 10 training subsets, we trained the five classifiers to derive five models; 3) we evaluated the accuracy of the 50 models (10 for each classifier corresponding to different training sample size) using the independent testing data; 4) we repeated the steps of 1) to 3) for 50 times, so that for each classifier and each sample size, we have 50 replicates. We then selected three out of the five classifiers based on high accuracy and robustness to sample size.

#### Integration of classifiers through Majority-vote and Bayesian-average data fusion methods

We further integrated the three best models based on the three classifiers selected in Part I and the full training dataset, via Majority-vote and Bayesian-average data fusion methods. Classifiers differ in learning algorithm and feature extraction, and can provide complementary information in classification. Therefore, data fusion of the results from multiple classifiers could potentially improve accuracy, effectiveness, and robustness in image classification, compared to individual classifiers [65]. The Majority-vote method is a simple data fusion based on hard classification and the class is defined by the one voted by the majority of multiple classifiers, i.e., at least 2 classifiers voted for the LULC class. The Bayesian average method is based on soft classification which takes the linear average of probabilities of multiple classifiers for each class (Eq 1). The final class is determined by the one having the highest average probability (Eq 2).

$$P(X\in C_{i})=\frac{1}{K}\sum_{k=1}^{K}P_{k}(X\in C_{i}),\;i=1,\cdots ,M$$ (1)

$$L(X)=\text{arg}\;\underset{i=1,\ldots ..,M}{\text{max}}(P(X\in C_{i}))$$ (2)

where, *P*_*k*_(*X* ∈ *C*_*i*_) is the probability belonging to class *i* determined by the *k* ^th^ classifier for pixel *X*; *M* and *K* are the total numbers of classes and classifiers, respectively; and *L*(*X*) is the final class for pixel *X* [65]. In this study, *K* = 3 and *M* = 9.

We evaluate the performance of the Majority-vote and the Bayesian-average integration methods by quantifying their classification accuracies on the testing data. For Bayesian-average integration, we also investigated the relationship between the integrated class membership probability and the classification accuracy for each class.

Finally we applied the integrated models to the entire APTBNC to obtain the LULC maps and corresponding class membership probability maps in 2013. For the Majority-vote integration, we evaluated its uncertainty by calculating the number of votes for each pixel. Number 3 indicates the consensus of all three classifiers, while number 1 indicates no common vote (large uncertainty). We further analyzed the uncertainties of the pixels with number 1 by calculating the proportions of LULC classes voted by each of the three classifiers, compared to their overall proportions in the study area, which will allow us to figure out the LULC types that are easily misclassified in this area. For the Bayesian-average integration, we evaluated its uncertainty through the corresponding class membership probability map. For the low-probability pixels indicating large uncertainty, we analyzed again the proportions of LULC classes versus their overall proportions in the study area, and compared with the corresponding results for the Majority-vote integration.

#### LULC changes in APTBNC between 2003 and 2013

To detect the decadal changes in LULC, we applied the Bayesian-average integrated classification based on RF, SVM, and WKNN for the years of 2003 and 2013. We then analyzed the decadal trend in each LULC type. We overlaid the two LULC maps to detect the changes in both location and extent. Finally we evaluated the conversions between different LULC types using the conversion matrix.

## Results

### Classification using five individual classifiers

#### Classification accuracy

The overall accuracies and Kappa statistics clearly showed that RF, SVM, and WKNN outperformed ANN and NB for any of the three input datasets (Table 1). Among the three input datasets, RF, SVM, and WKNN all achieved the highest overall accuracies with the input dataset C3 including the reflectances, VIs, phenology, and topographic information. The Kappa statistics for RF, SVM, and WKNN were 84.4%, 86.7%, and 85.5%, respectively, substantially higher than those obtained from the ANN (77.2%) and the NB (61.6%) classifiers for the input dataset C3. Therefore, we chose the RF, SVM, and WKNN classifiers and the input dataset C3 for further classification.

**Table 1:** Classification accuracies of five classifiers with three input datasets. (UA, user’s accuracy; PA, producer’s accuracy; OA, overall accuracy; C1, the growing-season statistics of reflectances, VIs and topographic variables; C2, phenology metrics and topographic variables; and C3, the combination of C1 and C2).

Input dataset	Classifier	Average UA (%)	Average PA (%)	OA (%)	Kappa (%)
C1	ANN	81.4	79.1	83.5	79.5
	NB	65.0	68.9	68.1	61.4
	RF	85.6	82.2	86.6	83.3
	SVM	86.9	84.3	88.2	85.3
	WKNN	86.7	83.8	87.4	84.3
C2	ANN	68.6	67.3	74.1	67.3
	NB	63.9	58.4	64.5	56.9
	RF	85.0	79.3	85.5	81.9
	SVM	83.3	79.9	84.6	80.8
	WKNN	82.2	78.0	83.3	79.2
C3	ANN	76.8	75.6	81.8	77.2
	NB	65.8	69.8	68.2	61.6
	RF	86.5	83.2	87.5	84.4
	SVM	87.2	84.9	89.3	86.7
	WKNN	87.2	83.9	88.3	85.5

For RF, SVM, and WKNN classifiers, the classification accuracies are high for forest, closed shrubland, grassland, agricultural land, urban area, and water, but relatively low for open shrubland and bare ground (Table 2). The two forest classes, agricultural land, and urban area have larger than 90% producer’s accuracies for any of the three classifiers. The omission error of grassland is about 14±1.8%, due to similar spectral characteristics among dense grass, crops, and closed shrubs. The omission error of closed shrubland is 13.6±3.0%, mainly because of the misclassification as grassland, open shrubland, deciduous forest, or cropland. Bare ground with 67.6±2.7% accuracy is sometimes confused with open shrubland. The class of open shrubland has the lowest accuracy and tends to be misrecognized as the closed shrubland or the bare ground.

**Table 2:** Confusion matrix for the classifiers of RF, SVM, and WKNN using the input dataset with all the predictor variables (UA, user’s accuracy; PA, producer’s accuracy; OA, overall accuracy; Kappa, Kappa statistics).

	Reference									
	AGRI	BARE	CLSH	DBLF	ENLF	GRAS	OPSH	URBN	WATR	UA
**RF**										
AGRI	548	5	10	0	0	19	9	2	0	92.4%
BARE	0	52	3	0	0	0	8	0	1	81.3%
CLSH	5	3	248	6	0	11	22	7	0	82.1%
DBLF	0	0	10	184	1	0	0	0	0	94.4%
ENLF	0	0	1	0	55	0	0	0	0	98.2%
GRAS	26	3	17	0	0	159	4	4	1	74.3%
OPSH	0	9	9	0	0	0	35	0	0	66.0%
URBN	4	2	1	0	0	0	2	151	4	92.1%
WATR	1	0	0	0	0	0	0	0	38	97.4%
**PA**	93.8%	70.3%	82.9%	96.8%	98.2%	84.1%	43.8%	92.1%	86.4%	
	Average PA = 83.2%, Average UA = 86.5%, OA = 87.5%, Kappa = 84.4%									
**SVM**										
AGRI	555	4	8	0	0	15	6	6	0	93.4%
BARE	2	48	1	0	0	0	10	0	1	77.4%
CLSH	3	5	264	11	0	7	17	3	1	84.9%
DBLF	0	0	6	178	0	0	0	0	0	96.7%
ENLF	0	0	1	0	55	0	0	0	0	98.2%
GRAS	18	4	11	1	0	166	6	2	1	79.4%
OPSH	1	11	6	0	0	1	40	0	0	67.8%
URBN	4	1	1	0	0	0	1	153	0	95.6%
WATR	1	1	1	0	1	0	0	0	41	91.1%
**PA**	95.0%	64.9%	88.3%	93.7%	98.2%	87.8%	50.0%	93.3%	93.2%	
	Average PA = 84.9%, Average UA = 87.2%, OA = 89.3%, Kappa = 86.7%									
**WKNN**										
AGRI	547	3	5	0	0	11	7	5	0	94.6%
BARE	2	50	3	0	0	0	10	0	0	76.9%
CLSH	12	6	263	11	0	16	16	7	4	78.5%
DBLF	0	0	7	179	0	1	0	0	0	95.7%
ENLF	0	0	0	0	56	0	0	0	0	100%
GRAS	19	1	11	0	0	161	4	3	1	80.5%
OPSH	1	12	9	0	0	0	43	0	0	66.2%
URBN	2	2	1	0	0	0	0	149	3	94.9%
WATR	1	0	0	0	0	0	0	0	36	97.3%
**PA**	93.7%	67.6%	88.0%	94.2%	100%	85.2%	53.8%	90.9%	81.8%	
	Average PA = 83.9%, Average UA = 87.2%, OA = 88.3%, Kappa = 85.5%									

#### Sensitivity of classification accuracy to training sample size

**Fig 3:**
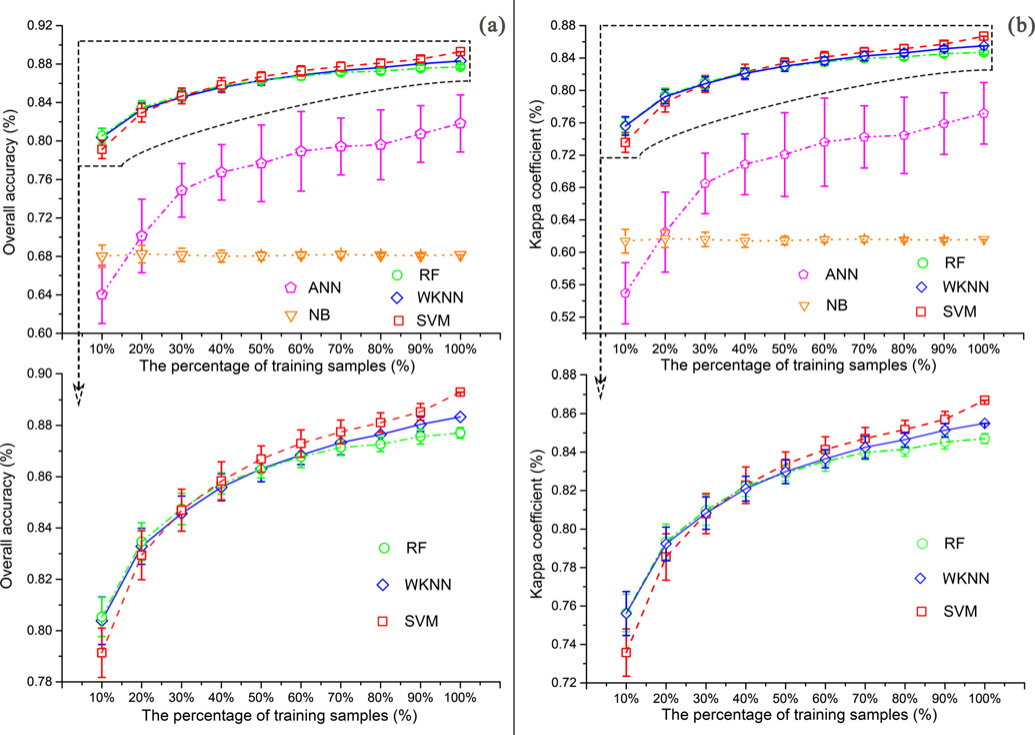
The overall accuracies (left panels) and Kappa statistics (right panels) for the five classifiers using variable training sample size. (dot, average accuracy statistics of the 50 random samples; error bar, one standard deviation away from the average; two figures below, zoomed from a and b, respectively, to highlight the trends for the RF, SVM, and WKNN).

The overall accuracies and Kappa statistics improved with the increase in training sample size (Fig 3). For a small sample size (i.e. 10% of training samples per class), the overall accuracies (Fig 3A) for RF, SVM, and WKNN were more than 79%, substantially higher than those of ANN (64%) and NB (68%). While the accuracies of RF, SVM, and WKNN increase with the sample size, their standard deviations decrease (Fig 3C and 3D). The differences of overall accuracies and Kappa statistics among RF, SVM, and WKNN become decreasing when the sample size increases from 10% to 40%, and then become increasing when from 50% to 100%.

The classification accuracies for RF, SVM, and WKNN are consistently greater than those of NB and ANN, and less sensitive to training sample size than ANN (Fig 3A and 3B), The overall accuracies for RF, SVM, and WKNN change less than 10% when the training sample size changes from 10 to 100% of training dataset. Moreover, it only causes a slightly lower overall accuracy of the three classifiers by 2±0.6%, compared to the maximums of the curves, when using 50 to 90% of training dataset. The overall accuracy and Kappa statistics for NB stay very low and are not sensitive to the sample size. NB assumes strong independent assumption of the feature variables so that only a small training sample size is needed to represent the feature space [66]. In reality, the multispectral variables are seldom independent. The low accuracy of ANN might be a result of inadequate size of the network.

### Integrated LULC mapping and uncertainty analysis

#### Majority-vote integrated LULC map and uncertainty analysis

The Majority-vote integrated classification improved the producer’s accuracy (Table 3) of agricultural land compared to all the three individual classifiers, and those of two forest classes, grassland, and urban area compared to two out of the three classifiers (Table 2). However, it did not for the bare ground and the open shrubland. The overall accuracy and Kappa statistics are higher than those of RF and WKNN, and close to those of SVM.

**Table 3:** Confusion matrix for the Majority-vote and the Bayesian-average integrated classifications based on the classifiers of RF, SVM, and WKNN. (UA, user’s accuracy; PA, producer’s accuracy; OA, overall accuracy; Kappa, Kappa statistics; UNCE, unclassified).

	Reference									
	AGRI	BARE	CLSH	DBLF	ENLF	GRAS	OPSH	URBN	WATR	UA
**Hard Integration using Majority vote**										
AGRI	558	2	8	0	0	11	8	4	0	94%
BARE	1	49	2	0	0	0	11	0	0	78%
CLSH	5	3	262	8	0	10	19	5	2	83%
DBLF	0	0	7	182	0	0	0	0	0	96%
ENLF	0	0	1	0	56	0	0	0	0	98%
GRAS	14	2	11	0	0	164	2	2	1	84%
OPSH	0	12	6	0	0	0	37	0	0	67%
URBN	3	1	0	0	0	0	0	152	1	97%
WATR	1	0	0	0	0	0	0	0	38	97%
UNCE	2	5	2	0	0	4	3	1	2	
**PA**	96%	66%	88%	96%	100%	87%	46%	93%	86%	
	*Average PA = 84*.*1%*, *Average UA = 88*.*4%*, *OA = 89*.*2%*, *Kappa = 86*.*5%*									
**Soft Integration using Bayesian average**										
AGRI	556	3	5	0	0	9	7	6	0	95%
BARE	1	53	3	0	0	0	11	0	0	78%
CLSH	3	2	265	8	0	11	20	3	2	84%
DBLF	0	0	7	182	0	0	0	0	0	96%
ENLF	0	0	0	0	56	0	0	0	0	100%
GRAS	17	3	9	0	0	169	5	2	1	82%
OPSH	1	11	9	0	0	0	37	0	0	64%
URBN	5	2	1	0	0	0	0	153	1	94%
WATR	1	0	0	0	0	0	0	0	40	98%
**PA**	95%	72%	89%	96%	100%	89%	46%	93%	91%	
	*Average PA = 85*.*7%*, *Average UA = 87*.*9%*, *OA = 89*.*9%*, *Kappa = 87*.*5%*									

**Fig 4:**
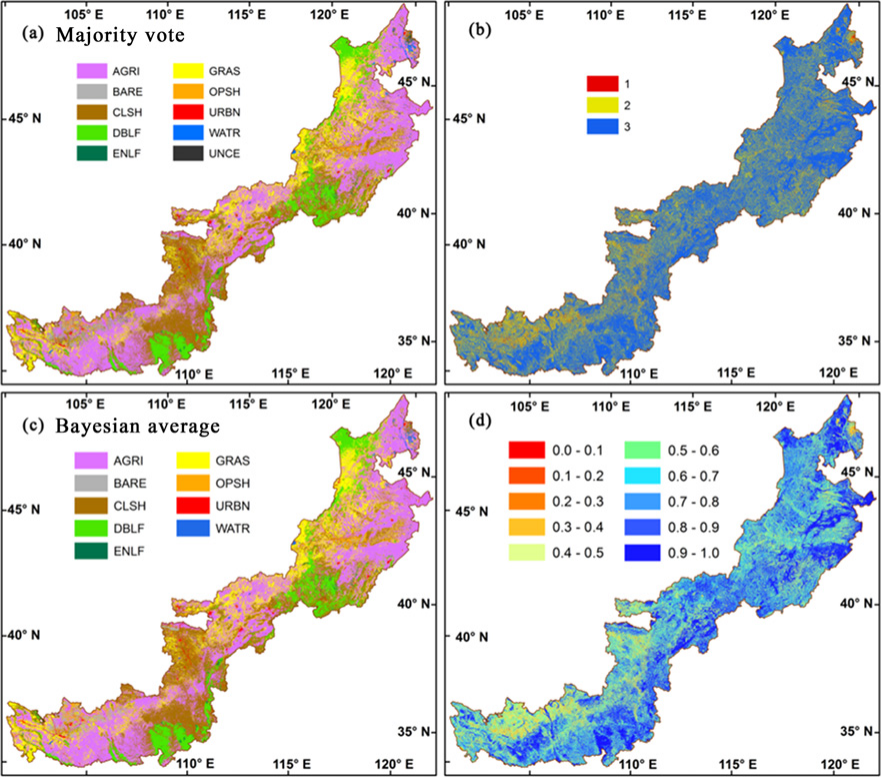
LULC maps in 2013 and their uncertainties using the Majority-vote integration (a & b) and the Bayesian-average integration (c & d) based on the RF, SVM, and WKNN classifiers. AGRI, agricultural land; BARE, bare ground; CLSH, closed shrubland; DBLF, deciduous broadleaved forest; ENLF, evergreen needle leaved forest; GRAS, grassland; OPSH, open shrubland; URBN, urban area; WATR, water; UNCE, unclassified; the legend of b, 3 indicates consensus vote from all 3 classifiers, 2 indicates 2 out of 3 classifiers having the same vote, and 1 indicates no common vote; the legend of d, class membership probability.

We applied the Majority-vote integrated classification to the whole study area of APTBNC for the year of 2013 (Fig 4A). The uncertainty of the resultant LULC map was assessed using the number of votes for the labeled class for each pixel (Fig 4B). About 69% of the region is labeled as 3, which indicates the overall consensus from all the three classifiers. About 28% of the region receives the same vote from 2 out of the 3 classifiers. Only less than 3% of the region could not reach any common vote, which is left as unclassified.

The unclassified pixels are mainly located in the southwest (Fig 4B), where agricultural lands, grasslands, and shrublands are interwoven (Fig 4A and Table 4). The ratio of proportion in unclassified pixels to its corresponding proportion in APTBNC for each class voted by the three classifiers can be used to detect those classes difficult to be recognized, i.e. those with the ratio much higher than 1. The classes of bare ground, open shrubland, urban area, and water have such ratios higher than 3, and therefore are difficult to identify in this area.

**Table 4:** Uncertainty analyses for the LULC maps of APTBNC in 2013 using the Majority-vote integration and the Bayesian-average integration. Numbers are proportions among unclassified pixels of Majority-vote integration or proportions among low membership probability pixels of Bayesian-average integration for each class; The ratio of the above proportion to its corresponding proportion in APTBNC is in the parenthesis; AGRI, agricultural land; BARE, bare ground; CLSH, closed shrubland; DBLF, deciduous broadleaved forest; ENLF, evergreen needle-leaved forest; GRAS, grassland; OPSH, open shrubland; URBN: urban area; WATR, water; and PRO, probability membership.

	AGRI	BARE	CLSH	DBLF	ENLF	GRAS	OPSH	URBN	WATR
unclassified pixels									
RF	28 (0.7)	3 (1.8)	19 (0.8)	3 (0.3)	1.6 (2.9)	41 (2.1)	3 (1.4)	2 (2.2)	0.1 (0.2)
SVM	33 (0.8)	7 (4.4)	15 (0.6)	5 (0.5)	0.7 (1.2)	16 (0.8)	13 (6.1)	6 (6.3)	5.5 (8.9)
WKNN	10 (0.3)	7 (4.7)	38 (1.6)	5 (0.5)	0.5 (0.9)	18 (1.0)	17 (8.0)	4 (3.9)	0.2 (0.3)
Mean	24 (0.6)	6 (**3.6)**	24 (1.0)	4 (0.4)	0.9 (1.7)	25 (1.3)	11 (**5.2)**	4 (**4.1)**	1.9 (**3.1)**
low membership probability pixels									
PRO < = 0.3	20 (0.5)	9 (4.8)	23 (1.0)	2 (0.2)	0.5 (0.8)	22 (1.1)	14 (5.8)	6 (4.7)	3.9 (6.0)
PRO < = 0.4	22 (0.5)	7 (**4.0**)	27 (1.1)	3 (0.3)	0.6 (0.9)	25 (1.3)	10 (**4.1)**	4 (**2.8)**	1.6 (**2.4)**

#### Bayesian-average integrated LULC map and uncertainty analysis

The Bayesian-average integrated classification greatly improved the producer’s accuracies compared to the three individual classifiers and the Majority-vote integration, especially for the classes of bare ground (72% versus 66% for Majority-vote) and grassland (Tables 2 and 3). The exception is for open shrubland, which is still largely confused with closed shrubland or bare ground. The overall accuracy and Kappa statistics achieve the highest of 89.9% and 87.5%, respectively.

**Fig 5:**
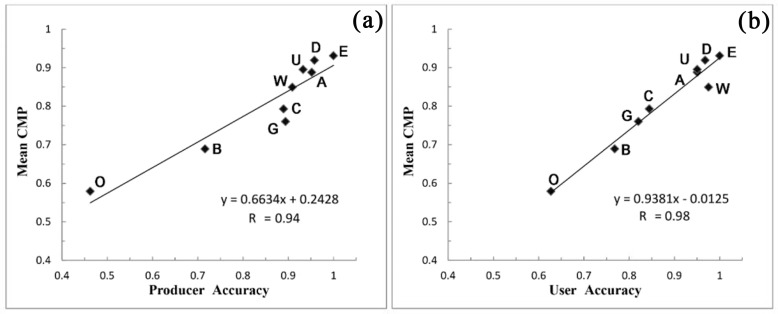
The means of integrated class membership probability (CMP) versus the classification accuracies (a. producer’s accuracy and b. user’s accuracy) for each LULC class. A, agricultural land; B, bare ground; C, closed shrubland; D, deciduous broadleaved forest; E, evergreen needle-leaved forest; G, grassland; O, open shrubland; U, urban area; and W, water.

The mean of integrated class membership probability for each class has statistically significant correlations with the corresponding classification accuracies, i.e. with correlation coefficients of 0.94 for producer’s accuracy and 0.98 for user’s accuracy (Fig 5).

We also applied the Bayesian-average integrated classification to APTBNC for the year of 2013 and evaluated the uncertainty of the map (Fig 4C and 4D). The method exploits the advantages of soft classification technique, especially in complex landscapes. The membership probability is larger than 0.5 for about 80% of the region. Only about 7.6% of the region has the probability lower than 0.4, which is mainly in the southwest (Fig 4D) and coincident with the unclassified pixels identified by the Majority-vote integration (Fig 4B). The high ratio of proportion in low membership probability area to its corresponding proportion in APTBNC for each class (Table 4) also points out that the bare ground and the open shrubland are hard to separate in this area.

### Decadal changes in LULC of APTBNC between 2003 and 2013

**Fig 6:**
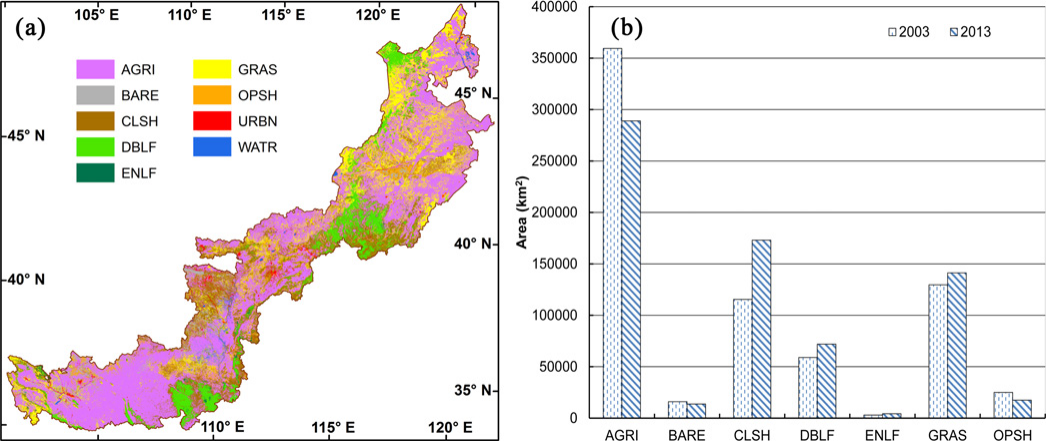
LULC map in 2003 using the Bayesian-average integrated classification (a) and distribution areas in 2003 and 2013 for each LULC type (b). AGRI, agricultural land; BARE, bare ground; CLSH, closed shrubland; DBLF, deciduous broadleaved forest; ENLF, evergreen needle-leaved forest; GRAS, grassland; and OPSH, open shrubland.

To detect the decadal changes in LULC, we applied the Bayesian-average integrated classification based on RF, SVM, and WKNN to the year of 2003 (Fig 6A) and evaluated the changes by comparing the map of 2003 to that of 2013 (Fig 4C). During this decade, closed shrubland and forests experienced significant expansions with 50% and 23% increase, respectively (Fig 6B). Grassland also expanded 9% in this period. With the expansions of closed shrubland, forests, and grassland, agricultural land shrank 20%, open shrubland decreased 30%, and bare ground reduced 15%.

The conversions between different LULC types in 2003–2013 revealed the details on the fact that the increases in closed shrubland, forest, and grassland are mainly from the decline in agricultural land (the major contributor), as well as the decrease in open shrubland and bare ground (Table 5). The net changes from AGRI are 34,971 into CLSH, 26,007 into GRAS, 2,825 into forest, 4,196 into bare ground, and 2,195 km^2^ into open shrubland. Open shrubland was mainly changed into closed shrubland (8,505 km^2^) and grassland (1,749 km^2^), but also received a net gain of 2,195 km^2^ from the abandoned agriculture. Bare ground was converted to closed shrubland (4,507 km^2^) and grassland (1,344 km^2^). In addition to the land converted from abandoned agriculture (2,825 km^2^), there are also a net of 7,195 of closed shrubland and a net of 4,392 km^2^ of grassland changed into forest through succession in this period. Meanwhile, there is a net of 13,617 km^2^ of grassland changed into closed shrubland, possibly also via succession.

**Table 5:** Land conversion matrix between 2003 and 2013 (km^2^). AGRI, agricultural land; BARE, bare ground; CLSH, closed shrubland; DBLF, deciduous broadleaved forest; ENLF, evergreen needle-leaved forest; GRAS, grassland; and OPSH, open shrub.

	2013						
2003	AGRI	BARE	CLSH	DBLF	ENLF	GRAS	OPSH
AGRI	236,821	6,421	43,901	2,583	592	60,392	4,870
BARE	2,225	4,670	4,810	0	0	1,824	2,219
CLSH	8,930	303	73,201	12,497	997	16,079	2,289
DBLF	336	0.1	5,985	50,959	535	1,098	0.8
ENLF	14	0	314	736	1,897	10	0.2
GRAS	34,385	479	29,696	5,219	281	56,823	1,469
OPSH	2,674	1,671	10,794	2	11	3,218	6,296

## Discussion

With rapid progress in remote sensing techniques, LULC products derived from mid-to-high resolution satellite imageries, such as MODIS and Landsat TM/ETM, have largely increased and thus provided key information in continuous monitoring of LULCC, modeling biogeochemical and hydrologic processes, simulating feedbacks to regional climate, and assessing impacts on biodiversity and ecosystem services. However, it is still a great challenge to produce accurate and reliable LULC maps over large areas with proper quality assessment, especially for those areas with complex landscapes. Feature variables and their spatiotemporal resolutions may be inadequate to discern the pattern and texture among the classes. And the uncertainty in the classification has to be explicitly analyzed before the classification result can be applied. Analysis with multiple classifiers has been proved more effective [65] than studies with individual classifies such as the applications of RF and SVM by Clark et al. [25] and Shao and Lunetta [67]. In this paper, we presented a robust LULC classification scheme by integrating multiple classifiers with posterior data fusion, which not only produces multiyear LULC maps with high accuracy, but also provides pixel-level uncertainties for further analyses.

Landsat TM/ETM+ spectral data, with finer spatial resolution than MODIS, are frequently adopted for LULC mapping in regional and recently global scales [68,69]. Although TM/ETM data provide more detailed spatial information, their low temporal resolution limits accurate LULC mapping, especially in time, due to cloud contamination, snow, lacking of images in appropriate season, etc. In our study, the higher temporal resolution of MODIS data is used to derive temporal-related features, such as growing-season statistics and phenology parameters, and thus to improve classification accuracy. In addition, high-frequency LULC maps benefit the investigation of abrupt and gradual LULC changes and provide complete perspective of LULCC dynamics, which is the key to evaluate the effectiveness of land use policies.

### Integrated data fusion methods in LULC classification and uncertainty analyses

We chose five common-used classifiers, of which RF, SVM, and WKNN achieved the highest accuracies and are robust to the changes in training sample size. We also compared the overall accuracy among three combinations of input variables. The overall accuracies and Kappa statistics improved when we add phenological parameters to the input dataset. The phenology information might help distinguish LULC types when phenology parameters are more distinctive among the LULC classes than within the LULC classes, e.g. evergreen versus deciduous [25].

In our study, the Bayesian-average integration achieved the highest accuracy than the individual classifiers and the Majority-vote integration. Beyond the advantage in high accuracy, its uncertainty map (Fig 4D) also conveys important information on pixel-level quality assessment, spatial variation in uncertainty, and key areas/LULC types for further analysis (Table 4).

LULC classification in large areas with mid-resolution can raise great challenges in quality assessment partly due to the fact that the sample points are mostly from spatially homogeneous areas, but there may exist some spatially heterogeneous pixels, i.e. mixed pixels, in reality [25,70]. Therefore, there may exist considerable spatial variation in uncertainty when applying to a large area. The pixel-level uncertainty map indexed by class membership probability, derived from the Bayesian-average data fusion, produces a means of assessing spatial variation in uncertainty. The high correlation (> 0.9, Fig 5) between the summarized membership probability and the PA’s or UA’s accuracy indicates that the pixel-level uncertainty map is in agreement with the traditional accuracy assessment.

The uncertainty map pinpoints the areas with high uncertainty in LULC mapping and allows us to determine the LULC types that are difficult to be distinguished. In this study, the southwestern area of APTBNC showed up with great uncertainty (Fig 4D), and the open shrubland is easily confused with the bare ground (Table 4). Special care should be taken for area with uncertainty when applying the classification results. Further recognition analysis may be prescribed for the uncertainty areas. Possible solutions may include building a synergy of MODIS with high resolution imagery (e.g. Landsat TM/ETM) or combining optical imagery with SAR (Synthetic Aperture Radar) and LiDAR (Light Detection and Ranging) imagers which can give a vertical domain observation to build 3-D land surface (e.g. the SAR data from the Advanced Land Observing Satellite and Sentinel 1).

**Fig 7:**
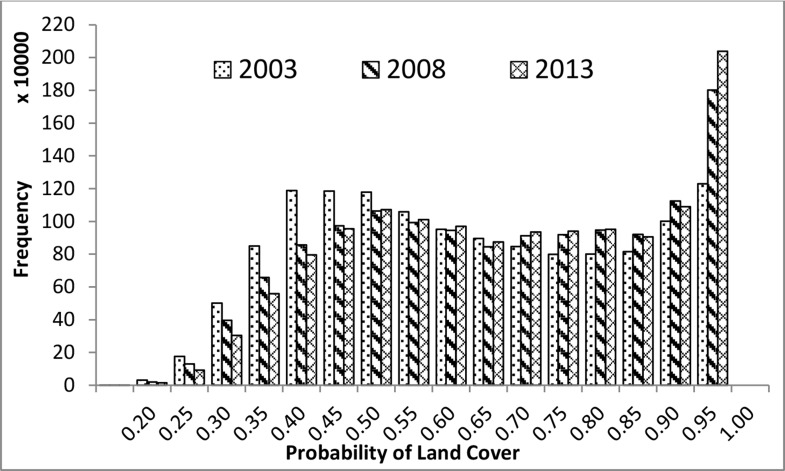
Histograms of the membership probability maps, derived from the Bayesian-average data fusion, for the years of 2003, 2008 and 2013 in APTBNC.

Multi-year LULC mapping is very important for the study of LULCC. However, it is prone to uncertainty when extrapolation to years without many training sample points derived from high-resolution images. Therefore, it is necessary to assess how reliable the LULC mapping is for multiple years. The pixel-level uncertainty map, explicitly derived from the Bayesian-average data fusion, meets such needs in multi-year LULC mapping. For instance, we applied the Bayesian-average data fusion in the LULC mapping of APTBNC for the years of 2003, 2008, and 2013. The histogram of class membership probability by our classification system (Fig 7) showed that most of the values are greater than 0.95 for the three years, thus having low uncertainty. The probabilities are higher for the years of 2008 and 2013, but a slightly lower for 2003, the year having the least samples. Hence we can check the robustness of the multi-year LULC mapping and assess the pixel-level reliability for each year using the uncertainty maps derived from the Bayesian-average data fusion.

### Policy change led to agricultural abandonment and woodland regrowth/expansion

**Fig 8:**
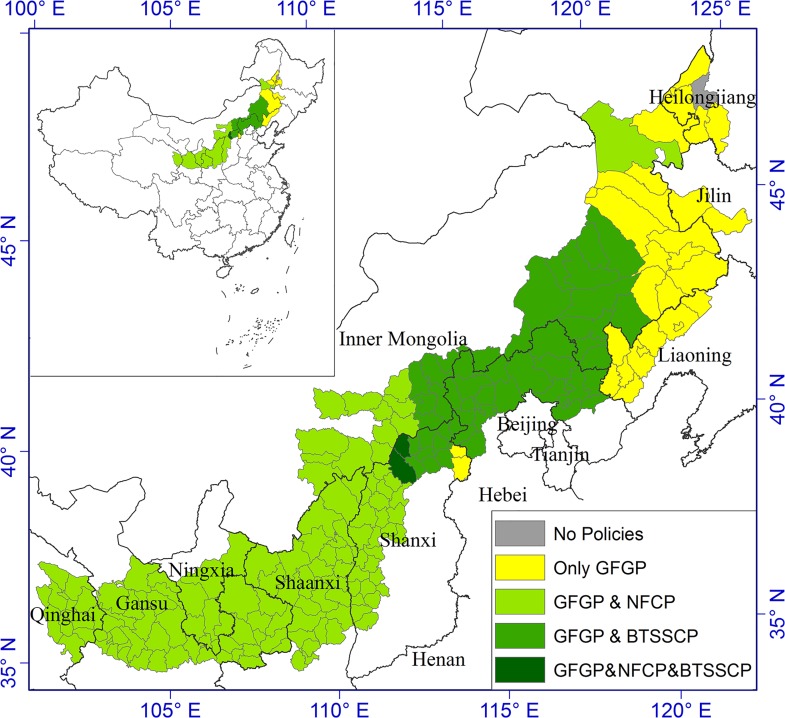
Three main ecological programs implemented by Chinese government in APTBNC. GFGP, Grain for Green Program; NFCP, Natural Forest Conservation Program; and BTSSCP, Beijing and Tianjin Sandstorm Source Control Project.

During 2003–2013, forests, closed shrublands, and grasslands all expanded in APTBNC with the shrinkages in croplands, bare ground, and open shrublands (Fig 6B). The general trends align with other small-scale studies in this region during the similar time period [13]. The significant decline in agricultural lands and expansion in forests, closed shrublands, and grasslands can be attributed to the national policy change aiming environmental protection, such as the implementation of six Key Forestry Programs (KFPs) on reforestation and ecological restoration [71]. APTBNC was covered by such programs due to its fragile and deteriorating ecosystems (Fig 8). The “Grain for Green” program, covering 99.5% of APTBNC, was implemented by Chinese central government in 1999 [13,37,72,73] to allow farmers to convert ill-conditioned farmland in steep slopes to forest/shrubland or grassland in the price of equivalent subsides and other tax relieves. The nationwide Natural Forest Conservation Program (NFCP), implemented in 1998 and covering 49.1% of APTBNC, protects and conserves forests mainly by building mountain closures and banned logging [72]. The Beijing and Tianjin Sandstorm Source Control Project (BTSSCP), implemented in 2000 and covering 27.4% of APTBNC, intends to reduce sandstorms in areas surrounding Beijing by conversion of cropland and vegetation regeneration [74,75].

Forests and closed shrublands increased in all the zones with the implementation of GFGP. The implementation of NFCP contributed to the highest increases (per area) in dense woody vegetation, including forests and closed shrublands, i.e. 14% of GFGP & NFCP and 10% of GFGP & NFCP & BTSSCP. Grassland increased in the zones covered by either NFCP or BTSSCP, and the BTSSCP brought about the highest increases (per area) in grassland, i.e. 6% of GFGP & BTSSCP and 11% of GFGP & NFCP & BTSSCP. The areas covered by all three programs reached the highest increase in combined dense woody and grass vegetation, i.e. 22% of GFGP & NFCP & BTSSCP converted to forests, closed shrublands, or grassland.

The national rural-to-urban migration might also be related to the LULCC in this region. Urban population in China increased from 18% to 53% in 1978–2012 and the National New-type Urbanization Plan released in 2014 set the urbanization rate as 1% per year until urban population reaches 60% in 2020 [76]. The consequent rural depopulation [77], with most emigrants to the developed coastal cities, might lead to agricultural abandonment. Our LULCC in APTBNC reflected the impacts of the policy changes, as the abandonment of agricultural land directly led to a net gain of 37,796 into closed shrubland and forests, as well as a net gain of 26,007 km^2^ into grassland (Table 5). The increased coverage of grass and woody plants would reduce soil erosion, improve mitigation of climate change, and enhance carbon sequestration in this region [37,38]. In addition to the policy change, the LULCC also depends on initial vegetation, climate, physical environment, implementation strength of policies, and other socioeconomic factors.

## Supporting Information

S1 Table Parameters of the classifiers used in building the classification models. (DOCX)
