# Supplementary material for: Decadal Trend in Agricultural Abandonment and Woodland Expansion in an Agro-Pastoral Transition Band in Northern China
Source: PLoS One. 2015 Nov 12;10(11):e0142113. doi: 10.1371/journal.pone.0142113 (PMC4643031; doi:10.1371/journal.pone.0142113)
Supplement: S1 Table — (DOCX) [file pone.0142113.s001.docx]

S1 Table Parameters of the classifiers used in building the classification models

| Classifier | Parameter | | |
| --- | --- | --- | --- |
| ANN | size | decay | rang |
|  | 10 | 5e-6 | 0.1 |
| NB | default |  |  |
| RF | mtry | ntree |  |
|  | 6 | 500 |  |
| SVM | kernel | cost | gamma |
|  | "radial" | 10 | 0.1 |
| WKNN | kernel | k | distance |
|  | "optimal" | 9 | 1 |
